# Impact of hypertension on liver fibrosis in patients with metabolic dysfunction-associated fatty liver disease

**DOI:** 10.3389/fmed.2025.1539283

**Published:** 2025-01-22

**Authors:** Zhifeng Gao, Huan Deng, Bowen Qin, Liang Bai, Jiangwei Li, Jian Zhang

**Affiliations:** ^1^Department of General Surgery Unit-4, The Second Affiliated Hospital of Xi’an, Jiaotong University Xi’an, Xi'an, Shaanxi, China; ^2^National and Local Joint Engineering Research Center of Biodiagnosis and Biotherapy, The Second Affiliated Hospital of Xi’an Jiaotong University, Xi'an, China

**Keywords:** hypertension, metabolic dysfunction-associated steatotic liver disease, significant fibrosis, advanced fibrosis, association

## Abstract

**Background:**

This study aims to evaluate the association between hypertension and the risk of fibrosis in metabolic dysfunction–associated steatotic liver disease (MASLD) patients, as well as to investigate the impact of hypertension on the progression of liver fibrosis within this population.

**Methods:**

We utilized data from the NHANES 2017 to March 2020. Multivariate logistic regression models were employed to control for sociodemographic and metabolic factors to determine the associations between hypertension, MASLD, and fibrosis.

**Results:**

Of the total cohort (*N* = 5,967) 57.92% had hypertension, 38.8% had MASLD, 25.88% had both MASLD and hypertension. Patients with MASLD were more likely to have hypertension (64.24% vs. 44.80%). There was a significant association between stage I (OR1.70, 95% CI: 1.15–2.53) and stage II hypertension (OR1.98, 95% CI: 1.38–2.85) and an increased risk of SF. After adjusting for multiple confounding factors, stage I (OR1.59, 95% CI: 1.09–2.24) and stage II hypertension (OR1.48, 95% CI: 1.06–2.06) remained significantly associated with the risk of SF. Patients with both MASLD and hypertension had higher rates of SF at 14.83% and AF at 7.47%. After adjusting for sociodemographic factors, those patients still had an 8.02-fold increased risk of SF (OR8.02, 95% CI: 4.47–14.39) and a 15.13-fold increased risk of AF (OR15.13, 95% CI: 7.09–32.3). Further adjustment for metabolic factors, those patients still had a significantly higher risk of SF (OR3.07, 95% CI: 1.83–5.14) and AF (OR4.01, 95% CI: 1.48–10.89).

**Conclusion:**

MASLD and hypertension are at risk for fibrosis, and the coexistence of the two has a more significant impact on the risk of fibrosis.

## 1 Introduction

Metabolic dysfunction–associated steatotic liver disease (MASLD) is a clinical and pathological syndrome characterized by diffuse fat deposition in liver cells, along with the gradual development of fatty degeneration and cellular inflammatory responses, in the absence of alcohol consumption or other obvious liver-damaging factors. It encompasses a series of liver lesions with varying degrees of damage and fibrosis (1). The global prevalence of MASLD is 32.4%, with men exhibiting a higher prevalence than women (2). MASLD is caused by multiple factors. It has been confirmed that genetics, metabolic syndrome (MS), and dietary patterns play key roles in the occurrence and progression of MASLD. Among these, metabolic syndrome is considered the strongest risk factor for the development of MASLD (1). The early symptoms of MASLD are often asymptomatic, and the disease progresses slowly. Without timely intervention, it can lead to liver fibrosis, cirrhosis, and even hepatocellular carcinoma (HCC). The occurrence and severity of fibrosis are the most important factors determining the outcome of MASLD (3, 4). The pathological basis of liver fibrosis is excessive deposition of extracellular matrix in liver cells, caused by chronic inflammation and fatty degeneration. The more severe the fibrosis, the higher the risk of death. Early clinical intervention offers a better benefit of positive clinical outcomes (5).

Hypertension is a clinical syndrome characterized by increased systemic arterial pressure, usually accompanied by functional or organic damage to organs such as the heart, brain, and kidneys. Hypertension is a multifactorial disease caused by the interaction of genetic and environmental factors. Studies have shown that hypertension is closely related to metabolic disorders and often coexists with obesity, hyperglycemia and dyslipidemia, forming metabolic syndrome (6). The study found that hypertension was significantly clustered in MASLD patients (7). Nearly or more than 50.0% of MASLD patients have hypertension (8). Similarly, about 50% of hypertensive patients will develop MASLD (9). Hypertension is associated with an increased risk of MASLD and is independently related to hepatic steatosis (10). Although previous studies have revealed a correlation between hypertension and MASLD, the relationship between hypertension and the risk of fibrosis in MASLD patients requires further exploration. This study aims to evaluate the relationship between hypertension and the risk of fibrosis in MASLD patients and to investigate the effect of hypertension on the progression of liver fibrosis in MASLD patients.

## 2 Methods

### 2.1 Study participants

The data used in this study are a nationally representative sample of pre-pandemic data from the National Health and Nutrition Examination Survey (NHANES) 2017 to March 2020, which is publicly available in the NHANES database.^1^ The protocol for the NHANES study was authorized by the National Center for Health Statistics (NCHS) Research Institutional Review Board. Informed consent was obtained from all NHANES participants.

### 2.2 Definition of hypertension and MASLD

Blood pressure in NHANES was calculated as the average of systolic blood pressure (SBP) and diastolic blood pressure (DBP) calculated from three consecutive measurements, each taken 60 s apart. According to the 2017 American Heart Association/American College of Cardiology (AHA/ACC) guidelines for the classification of hypertension, individuals with SBP ≥130 mmHg and/or DBP ≥80 mmHg were defined as having hypertension (11). Liver stiffness was measured by FibroScan® which uses the vibration controlled transient elastography (VCTETM) to derive liver stiffness. The device also simultaneously measures the ultrasound attenuation related to the presence of hepatic steatosis and records the controlled attenuation parameter (CAPTM) as the indicator of the degree of hepatic steatosis. Liver stiffness measurements range from 1.6 to 75 kPa, with higher values indicating more severe fibrosis. CAP values range from 100 to 400 dB/m, with higher values indicating more liver fat content. The diagnostic criteria for MASLD are a median CAP score ≥ 285 dB/m, which has a sensitivity of 80% and a specificity of 77% for detecting hepatic steatosis (12), excluding other chronic liver diseases and excessive drinking. Significant fibrosis (SF) and advanced fibrosis (AF) were defined as liver stiffness ≥8.0 kPa and ≥ 13.1 kPa, respectively (13).

### 2.3 Other covariates

We collected information on demographic characteristics and lifestyle. Smoking status was categorized as current smokers, former smokers, and never smokers, based on whether participants had smoked fewer than 100 cigarettes in their lifetime or whether they were current smokers. BMI was calculated as weight (kg) / height (m^2^). Diabetes was defined as a previous history of diabetes, an HbA1c level ≥ 6.5%, or a fasting blood glucose level ≥ 126 mg/dL (7.0 mmol/L).

### 2.4 Statistical analysis

Baseline data were presented as weighted means with standard errors (continuous variables) and frequencies with weighted percentages (categorical variables), and the differences between the groups were compared using the Kruskal Wallis test for continuous variables and the Rao-Scott chi-square test for categorical variables to determine differences in demographic and clinical characteristics between the comparison groups. Multivariate logistic regression models were used to control for sociodemographic and metabolic factors to determine the associations between hypertension and MASLD and fibrosis. Model 1 did not include any covariate adjustments; Model 2 included adjustments for age, sex, race, education level, income, marital status, smoking, and physical activity; Model 3 included additional adjustments for Glycosylated hemoglobin, Body mass index (BMI), High-density lipoprotein (HbA1c), Alanine Aminotransferase (ALT), Aspartate Aminotransferase (AST), Albumin (ALB), Gamma-Glutamyl Transferase (GGT), total bilirubin, total cholesterol, and triglycerides based on Model 2. The results were presented as odds ratios (ORs) and 95% confidence intervals (CIs). Given the complex sampling of NHANES, we took weights into account in the statistical analyses. All analyses were performed using the “survey” package in R 4.2.2. Statistical significance was determined by two-sided *p* < 0.05.

## 3 Results

### 3.1 Clinical and demographic characteristics of the study population

**Figure 1:**
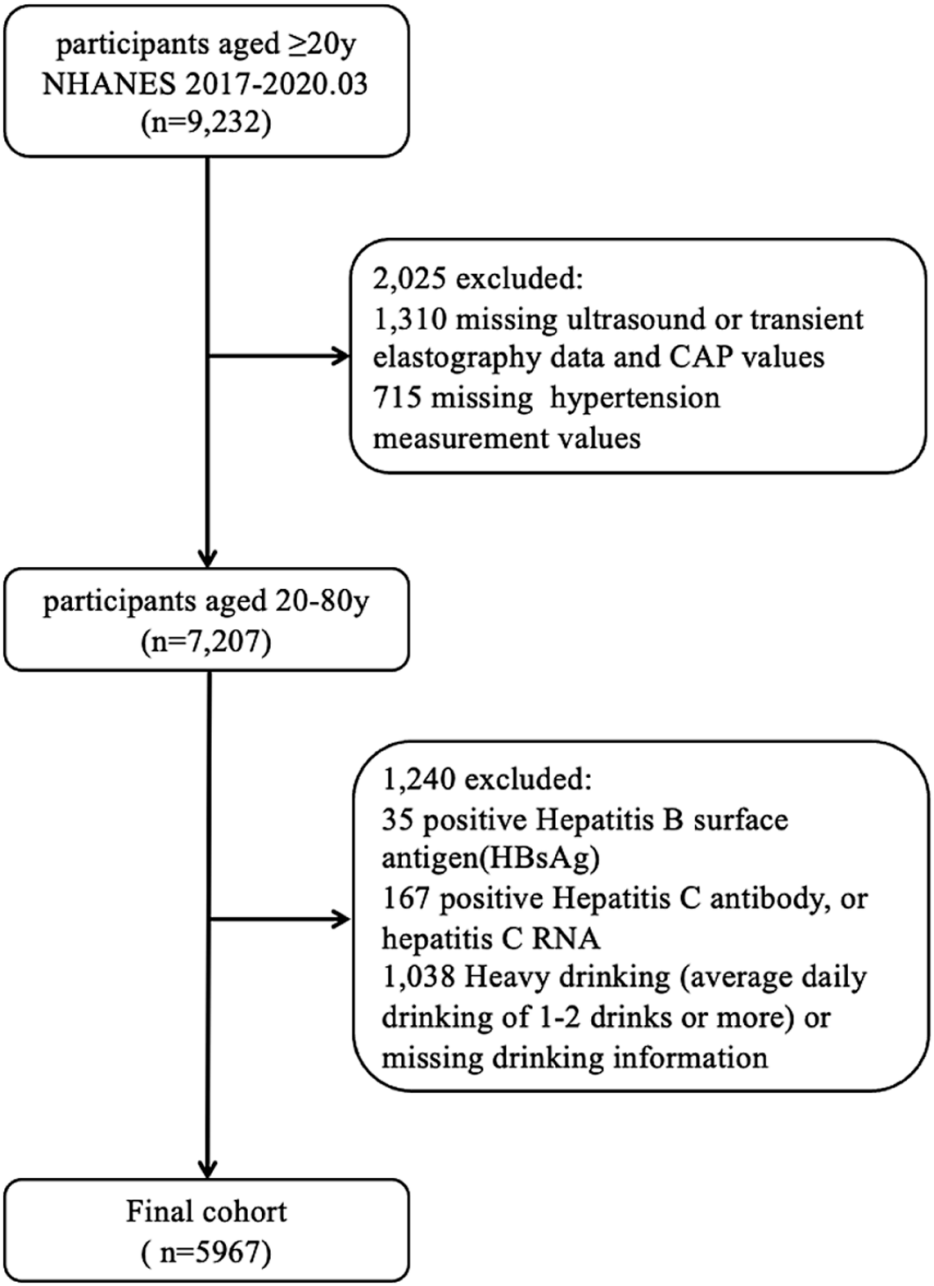
Flowchart of participant selection.

Among the initial 15,560 participants, 9,232 were aged 20 years or older. A total of 2,025 participants were excluded due to a lack of ultrasound or transient elastography data or hypertension measurements data. An additional 1,073 participants were excluded for the following reasons: 35 tested positive for hepatitis B surface antigen, 167 tested positive for hepatitis C antibodies or HCV RNA, and 1,038 were heavy drinkers (average daily drinking of 1–2 drinks or more) or had missing information on alcohol consumption. The final study cohort consisted of 5,967 participants (Figure 1).

### 3.2 Prevalence and associations of MASLD and hypertension

Among the study cohort, prevalence of MASLD was 38.80%, and among those with MASLD, 13.18% had SF, 7.38% had AF. Demographic and clinical characteristics of MASLD patients with and without MASLD are demonstrated in Table 1. Compared to individuals with non-MASLD, individuals with MASLD were more likely to have hypertension (64.24% vs. 44.80%) and diabetes (25.79% vs. 7.10%), as well as an older weighted mean age (50.48 vs. 45.82), a higher proportion of males (58.18% vs. 45.04%), a higher BMI (33.39 vs. 26.58), higher DBP (124.33 vs. 119.53) and SPB (76.88 vs. 72.18) levels (all *p* < 0.05) (Table 1).

**Table 1:** Demographic and clinical characteristics of the participants with and without NAFLD.

Characteristics	Non-NAFLD (*n* = 3,625)	NAFLD (*n* = 2,315)	*P*-value
Age (years)	45.82 (44.38–47.25)	50.48 (48.92–52.04)	0.00
Sex, *n* (%)	60.75%	38.80%	0.00
Male	45.04%	58.18%	
Female	54.96%	41.82%	
Race/ethnicity, *n* (%)			0.00
Mexican American	6.05%	11.65%	
Other Hispanic	7.71%	6.64%	
Non-Hispanic White	65.77%	64.60%	
Non-Hispanic Black	12.14%	8.45%	
Other Race-Including Multi-Racial	8.34%	8.66%	
Education level, *n* (%)			0.00
Less than high school	8.51%	10.03%	
High school or equivalent	24.88%	29.72%	
College or above	66.61%	60.24%	
Family poverty income ratio	2.59 (2.55–2.62)	2.6 (2.56–2.63)	0.89
Married, %	58.54%	68.18%	0.00
Diabetes, *n* (%)			0.00
Yes	7.10%	25.79%	
No	92.90%	74.21%	
Smoking status, *n* (%)			0.01
Current	17.34%	15.88%	
Never	58.31%	52.73%	
Former	24.36%	31.39%	
Weekly minutes	1372.34 (1279.27–1465.4)	1211.89 (1122.03–1301.75)	0.01
BMI	26.58 (26.3–26.86)	33.39 (32.87–33.91)	0.00
Non-hypertension	55.20%	35.77%	0.00
Hypertension, *n* (%)	44.80%	64.24%	0.00
Elevated	14.55%	15.14%	
Stage 1	16.38%	27.21%	
Stage 2	13.87%	21.89%	
SBP	119.53 (118.65–120.4)	124.33 (123.32–125.33)	0.00
DBP	72.18 (71.61–72.75)	76.88 (76.3–77.46)	0.00
Median stiffness (kPa)	4.99 (4.86–5.12)	7.38 (6.89–7.87)	0.00
Significant Fibrosis (%)	2.89%	13.18%	0.00
Advanced Fibrosis (%)	0.85%	7.38%	0.00
Median CAP (dB/m)	225.75 (223.98–227.52)	331.26 (328.85–333.68)	0.00
Fasting glucose (mg/dL)	5.66 (5.61–5.71)	6.69 (6.53–6.84)	0.00
HbA1c (%)	5.46 (5.43–5.5)	6 (5.93–6.08)	0.00
HDL-cholesterol (mg/dL)	57.14 (56.23–58.05)	47.01 (46.26–47.76)	0.00
LDL-cholesterol (mg/dL)	108.9 (106.91–110.89)	110.55 (107.52–113.58)	0.06
hs-CRP (mg/L)	3 (2.6–3.41)	4.95 (4.52–5.38)	0.00
ALT (U/L)	19.07 (18.52–19.62)	28.16 (26.93–29.38)	0.00
ALB (g/dL)	4.15 (4.13–4.17)	4.07 (4.05–4.1)	0.00
AST (U/L)	20.26 (19.88–20.63)	23.06 (22.46–23.66)	0.00
GGT (IU/L)	23.56 (22.24–24.88)	36.45 (34.7–38.21)	0.00
Total bilirubin (mg/dL)	0.48 (0.46–0.49)	0.45 (0.43–0.48)	0.09
Total cholesterol (mg/dL)	186.34 (183.87–188.81)	190.57 (187.05–194.09)	0.01
Triglycerides (mg/dL)	115.49 (111.11–119.88)	181.78 (174.94–188.61)	0.00
Uric acid (mg/dL)	5.06 (5–5.13)	5.86 (5.75–5.97)	0.00

In the study cohort, the prevalence of hypertension was 57.92%, the weighted mean age was 52.53 years, and 58% were male. Compared to individuals without hypertension, those with hypertension had higher levels of median stiffness (6.27 vs. 5.46) and median CAP (279.77 vs. 249.29), and higher prevalence of SF (8.95% vs. 4.32%), AF (4.13% vs. 2.38%), and MASLD (46.08% vs. 27.86%). In addition, compared to individuals with non-hypertension, individuals with hypertension had higher BMI (30.06 vs. 28.1) and higher rates of diabetes (18.05% vs. 9.77%) (Table 2). Further stratification by hypertension severity showed that the prevalence of SF was significantly different between groups (*p* < 0.05), increasing from 4.32% in the non-hypertension group to 10.70% in hypertension grade II. Median CAP increased from 249.29 dB/m in the non-hypertension group to 284.13 dB/m in hypertension grade II (*p* < 0.05) (Supplementary Table S1).

**Table 2:** Demographic and clinical characteristics of the participants with and without hypertension.

Characteristics	Non-Hypertension (*n* = 2,511)	Hypertension (*n* = 3,456)	*P*-value
Age (years)	42.17 (40.83–43.5)	52.53 (50.95–54.1)	0.00
Sex, *n* (%)	42.08%	57.92	0.00
Male	41.21%	58.00%	
Female	58.79%	42.00%	
Race/ethnicity, *n* (%)			0.00
Mexican American	9.07%	7.27%	
Other Hispanic	7.63%	7.01%	
Non-Hispanic White	66.18%	64.55%	
Non-Hispanic Black	9.08%	12.32%	
Other Race - Including Multi-Racial	8.03%	8.85%	
Education level, *n* (%)			0.00
Less than high school	7.48%	10.55%	
High school or equivalent	24.05%	29.12%	
College or above	68.46%	60.33%	
Family poverty income ratio	2.58 (2.55–2.62)	2.6 (2.57–2.64)	0.31
Married, %	59.44%	64.62%	0.00
Diabetes, *n* (%)			0.00
Yes	9.77%	18.05%	
No	90.23%	81.95%	
Smoking status, *n* (%)			0.00
Current	17.02%	16.58%	
Never	60.80%	52.01%	
Former	22.18%	31.40%	
Weekly minutes	1389.47 (1271.49–1507.46)	1241.45 (1161.78–1321.12)	0.02
BMI	28.1 (27.63–28.57)	30.06 (29.71–30.41)	0.00
SBP	108.32 (107.75–108.89)	133.29 (132.36–134.22)	0.00
DBP	67.28 (66.84–67.72)	80.07 (79.41–80.72)	0.00
Median stiffness (kPa)	5.46 (5.22–5.71)	6.27 (5.99–6.54)	0.00
Significant Fibrosis (%)	4.32%	8.95%	0.00
Advanced Fibrosis (%)	2.38%	4.13%	0.00
Median CAP (dB/m)	249.29 (245.02–253.55)	279.77 (276.92–282.61)	0.00
<285 dB/m (%)	72.14%	53.92%	0.00
≥285 dB/m (%)	27.86%	46.08%	0.00
Fasting glucose (mg/dL)	5.77 (5.7–5.84)	6.29 (6.19–6.4)	0.00
HbA1c (%)	5.5 (5.47–5.53)	5.82 (5.76–5.87)	0.00
HDL-cholesterol (mg/dL)	54.27 (53.25–55.28)	52.52 (51.49–53.55)	0.00
LDL-cholesterol (mg/dL)	106.91 (104.6–109.22)	111.92 (109.24–114.6)	0.00
hs-CRP (mg/L)	3.74 (3.37–4.11)	3.72 (3.38–4.05)	0.00
ALT (U/L)	20.07 (19.36–20.78)	24.67 (23.79–25.54)	0.00
ALB (g/dL)	4.13 (4.1–4.15)	4.11 (4.09–4.14)	0.15
AST (U/L)	20.26 (19.69–20.82)	22.27 (21.91–22.63)	0.00
GGT (IU/L)	23.3 (21.95–24.64)	33.05 (31.36–34.73)	0.00
Total bilirubin (mg/dL)	0.46 (0.44–0.48)	0.47 (0.45–0.49)	0.19
Total cholesterol (mg/dL)	183.49 (180.32–186.65)	192 (188.97–195.03)	0.00
Triglycerides (mg/dL)	127.09 (119.6–134.58)	152.36 (147.69–157.04)	0.00
Uric acid (mg/dL)	5.12 (5.03–5.2)	5.59 (5.52–5.65)	0.00

### 3.3 Association between hypertension and severity of fibrosis

The results of the multivariate regression analysis indicated a significant association between stage I hypertension [OR: 1.70 (95% CI, 1.15–2.53, *p* = 0.05)] and stage II hypertension [OR: 1.98 (95% CI, 1.38–2.85, *p* < 0.01)] and an increased risk of SF when confounding factors were not adjusted. After adjusting for age, sex, race, income, education, marital status, smoking, and physical activity, a significant association remained between stage I hypertension [OR: 1.62 (95% CI, 1.1–2.4, *p* = 0.02)] and stage II [OR = 1.67, 95% CI (1.13–2.46), *p* = 0.01] in relation to the risk of SF when compared to patients without hypertension. In further adjustments that accounted for multiple confounding factors such as BMI, diabetes, and liver enzymes, stage I hypertension [OR = 1.59, 95% CI (1.09–2.24), *p* = 0.01)] and stage II hypertension [OR = 1.48, 95% CI, 1.06–2.06, *p* = 0.02] remained significantly associated with the risk of SF (Table 3). However, an increased risk for AF was not observed.

**Table 3:** Multivariable odd ratio of hypertension for significant and advanced fibrosis.

	Model 1 OR (95% CI)	*P*-value	Model 2 OR (95% CI)	*P*-value	Model 3 OR (95% CI)	*P*-value
Significant fibrosis						
Non-Hypertension	Reference		Reference		Reference	
Elevated	1.3 (0.77–2.19)	0.33	1.17 (0.68–2.02)	0.57	1.03 (0.58–1.84)	0.92
Stage 1	1.7 (1.15–2.53)	0.01	1.62 (1.1–2.4)	0.02	1.59 (1.12–2.26)	0.01
Stage 2	1.98 (1.38–2.85)	0.00	1.67 (1.13–2.46)	0.01	1.48 (1.06–2.06)	0.02
Advanced fibrosis						
Non-Hypertension	Reference		Reference		Reference	
Elevated	1.13 (0.49–2.6)	0.78	0.96 (0.41–2.27)	0.93	0.89 (0.38–2.09)	0.79
Stage 1	1.54 (0.88–2.7)	0.13	1.38 (0.81–2.36)	0.24	1.44 (0.85–2.43)	0.17
Stage 2	1.12 (0.74–1.69)	0.61	0.94 (0.58–1.53)	0.81	0.95 (0.64–1.4)	0.78

Model 1: adjusted for: none. Model 2: adjusted for age, sex, race, income, education, marital status, smoking, and physical activity. Model 3: adjusted for Model 2 + diabetes, HbA1c, BMI, HDL-cholesterol, ALT, AST, ALB, GGT, total bilirubin, total cholesterol, triglycerides. OR, Odds Ratio; CI, confidence interval; BMI: Body mass index; HbA1c: Glycosylated hemoglobin; HDL: High-density lipoprotein; ALT: Alanine Aminotransferase; AST: Aspartate Aminotransferase; ALB: Albumin; GGT: Gamma-Glutamyl Transferase.

### 3.4 Association of MASLD and hypertension with severity of fibrosis

We assessed the impact of hypertension on fibrosis by stratifying the study population into four subgroups according to the presence of MASLD and hypertension. Approximately 25.88% of the study group had both MASLD and hypertension. Compared to individuals with MASLD alone or hypertension alone, those with both MASLD and hypertension had higher rates of SF at 14.83% and AF at 7.47%. In this context, while SF and AF were present 10.23 and 7.22% of patients with MASLD alone, and 3.92 and 1.27% in patients with hypertension alone (Supplementary Table S2).

Multivariate logistic regression analysis showed that after adjusting for multiple confounding factors such as age, sex, race, and education, marital status, smoking, and physical activity, compared to individuals with neither MASLD nor hypertension, individuals with MASLD alone had a 5.62-fold increased risk of SF [OR: 5.62 (95% CI, 3.15–10.02, *p* < 0.05)] and a 15.54-fold increased risk of AF [OR: 15.81 (95% CI, 7.72–31.29, *p* < 0.05)]. While patients with both MASLD and hypertension had an 8.02-fold increased risk of SF [OR: 8.02 (95% CI, 4.47–14.39, *p* < 0.05)] and a 15.13-fold increased risk of AF [OR: 15.13 (95% CI, 7.09–32.3, *p* < 0.05)]. Further adjustment for multiple metabolic factors such as BMI, diabetes, glucose, and liver enzymes, patients with both MASLD and hypertension still had a significantly higher risk of SF [OR: 3.07 (95% CI, 1.83–5.14, *p* < 0.05)] and AF [OR: 4.01 (95% CI, 1.48–10.89, *p* = 0.01)] (Table 4).

**Table 4:** Multivariable odd ratio of NAFLD and hypertension for significant and advanced fibrosis.

	Model 1 OR (95% CI)	*P*-value	Model 2 OR (95% CI)	*P*-value	Model 3 OR (95% CI)	*P*-value
Control	Reference		Reference		Reference	
Hypertension only	1.97 (1.21–3.2)	0.01	1.69 (1.01–2.84)	0.05	1.3 (0.78–2.17)	0.31
NAFLD only	5.91 (3.4–10.26)	0.00	5.62 (3.15–10.02)	0.00	2.13 (1.24–3.64)	0.01
NAFLD and Hypertension	9.1 (5.14–16.09)	0.00	8.02 (4.47–14.39)	0.00	3.07 (1.83–5.14)	0.00
Advanced fibrosis						
Control	Reference		Reference		Reference	
Hypertension only	2.53 (1.01–6.3)	0.05	2.24 (0.82–6.09)	0.11	1.73 (0.51–2.17)	0.38
NAFLD only	16.5 (8.12–33.52)	0.00	15.54 (7.72–31.29)	0.00	3.94 (1.38–11.25)	0.01
NAFLD and Hypertension	18.12 (8.54–38.48)	0.00	15.13 (7.09–32.3)	0.00	4.01 (1.48–10.89)	0.01

Model 1: adjusted for: none. Model 2: adjusted for age, sex, race, income, education, marital status, smoking, and physical activity. Model 3: adjusted for Model 2 + diabetes, HbA1c, BMI, HDL-cholesterol, ALT, AST, ALB, GGT, total bilirubin, total cholesterol, triglycerides. OR, Odds Ratio; CI, confidence interval; BMI: Body mass index; HbA1c: Glycosylated hemoglobin; HDL: High-density lipoprotein; ALT: Alanine Aminotransferase; AST: Aspartate Aminotransferase; ALB: Albumin; GGT: Gamma-Glutamyl Transferase.

## 4 Discussion

This study showed that patients with MASLD had a higher incidence of hypertension. Patients with hypertension had a higher incidence of SF and AF. Multivariate regression analysis showed that patients with both MASLD and hypertension had a higher risk of SF and AF compared with patients with only MASLD or only hypertension, and this risk remained even after adjusting for metabolic markers.

The accumulation of extracellular matrix in the liver leads to progressive fibrosis, cirrhosis, portal hypertension, and liver failure, which is the main cause of liver-related mortality in patients with MASLD (1). Systemic hypertension is closely associated with the progression of fibrosis (14). Studies have shown that 50% of hypertensive patients have MASLD (15) which is consistent with our findings. MASLD is also associated with arterial stiffness, myocardial remodeling, kidney disease, and heart failure (16–18). Hypertension is a strong indicator of increased risk of MASLD (10, 19). Study suggests that controlling blood pressure in non-obese hypertensive patients may be associated with a reduced risk of developing MASLD (20). In fact, liver disease is only the third leading cause of death in MASLD patients, after cardiovascular disease and malignant tumors (21, 22). Patients with MASLD are twice as likely to die from cardiovascular disease as from liver disease, which is primarily associated with shared risk factors such as diabetes, systemic hypertension, and obesity (1). Metabolic syndrome is an important driver of adverse cardiovascular events and total mortality in patients with MASLD (23, 24).

Although this study revealed the important effect of hypertension on liver fibrosis in patients with MASLD, there are still some limitations. First, this study was cross-sectional in design and could not clarify the causal relationship; second, we failed to consider other potential genetic or environmental factors, such as diet and lifestyle, which may have an important influence on the relationship between MASLD and hypertension. Future studies should include longitudinal data and a wider range of risk factors to further clarify the complex mechanism of hypertension on liver fibrosis in patients with MASLD.

## 5 Conclusion

In summary, our data shows that MASLD and hypertension are at risk for liver fibrosis, and the coexistence of the two has a more significant impact on the risk of fibrosis. This study highlights the interesting association between hypertension and fibrosis in the context of MASLD, providing valuable insights for the clinical management of patients with MASLD and hypertension.

## Transparency statement

The lead author affirms that this manuscript is an honest, accurate, and transparent account of the study being reported; that no important aspects of the study have been omitted; and that any discrepancies from the study as planned (and, if relevant, registered) have been explained.

## Data Availability

The original contributions presented in the study are included in the article/Supplementary material, further inquiries can be directed to the corresponding author.

## Footnotes

^1^ https://wwwn.cdc.gov/nchs/nhanes/Default.aspx

## References

[1] Friedman SL Neuschwander-Tetri BA Mary R Sanyal AJ. Mechanisms of Nafld development and therapeutic strategies. Nat Med. (2018) 24:908–22. doi: 10.1038/s41591-018-0104-9, PMID: 29967350 PMC6553468

[2] Riazi K Azhari H Charette JH Underwood FE King JA Afshar EE. The prevalence and incidence of Nafld worldwide: a systematic review and Meta-analysis. Lancet Gastroenterol Hepatol. (2022) 7:851–61. doi: 10.1016/s2468-1253(22)00165-0, PMID: 35798021

[3] Angulo P Kleiner DE Dam-Larsen S Adams LA Bjornsson ES Charatcharoenwitthaya P. Liver fibrosis, but no other histologic features, is associated with long-term outcomes of patients with nonalcoholic fatty liver disease. Gastroenterology. (2015) 149:389–97.e10. doi: 10.1053/j.gastro.2015.04.043, PMID: 25935633 PMC4516664

[4] Hagström H Nasr P Ekstedt M Hammar U Stål P Hultcrantz R. Fibrosis stage but not Nash predicts mortality and time to development of severe liver disease in biopsy-proven Nafld. J Hepatol. (2017) 67:1265–73. doi: 10.1016/j.jhep.2017.07.027, PMID: 28803953

[5] Heyens LJM Busschots D Koek GH Robaeys G Francque S. Liver fibrosis in non-alcoholic fatty liver disease: from liver biopsy to non-invasive biomarkers in diagnosis and treatment. Front Med. (2021) 8:615978. doi: 10.3389/fmed.2021.615978, PMID: 33937277 PMC8079659

[6] Alberti KG Eckel RH Grundy SM Zimmet PZ Cleeman JI Donato KA. Harmonizing the metabolic syndrome: a joint interim statement of the international diabetes federation task force on epidemiology and prevention; National Heart, Lung, and Blood Institute; American Heart Association; world heart federation; international atherosclerosis society; and International Association for the Study of obesity. Circulation. (2009) 120:1640–5. doi: 10.1161/circulationaha.109.192644 19805654

[7] Ryoo JH Suh YJ Shin HC Cho YK Choi JM Park SK. Clinical association between non-alcoholic fatty liver disease and the development of hypertension. J Gastroenterol Hepatol. (2014) 29:1926–31. doi: 10.1111/jgh.12643, PMID: 24910023

[8] Kirovski G Schacherer D Wobser H Huber H Hellerbrand C. Prevalence of ultrasound-diagnosed non-alcoholic fatty liver disease in a hospital cohort and its association with anthropometric, biochemical and sonographic characteristics. Int J Clin Exp Med. (2010) 48:560. doi: 10.1055/s-0029-1246560, PMID: 20827318 PMC2929946

[9] Golabi P Stepanova M Pham HT Cable R Rafiq N Bush H. Non-alcoholic Steatofibrosis (Nasf) can independently predict mortality in patients with non-alcoholic fatty liver disease (Nafld). BMJ Open Gastroenterol. (2018) 5:e000198. doi: 10.1136/bmjgast-2018-000198, PMID: 29607054 PMC5873539

[10] Yuan M He J Hu X Yao L Chen P Wang Z. Hypertension and Nafld risk: insights from the Nhanes 2017–2018 and Mendelian randomization analyses. Chin Med J. (2023) 137:457–64. doi: 10.1097/CM9.0000000000002753, PMID: 37455323 PMC10876227

[11] Whelton PK Carey RM Aronow WS Casey DE Jr Collins KJ Dennison Himmelfarb C. 2017 Acc/Aha/Aapa/Abc/Acpm/Ags/Apha/ash/Aspc/Nma/Pcna guideline for the prevention, detection, evaluation, and Management of High Blood Pressure in adults: executive summary: a report of the American College of Cardiology/American Heart Association task force on clinical practice guidelines. Circulation. (2018) 138:e426–83. doi: 10.1161/cir.0000000000000597 30354655

[12] Siddiqui MS Vuppalanchi R Van Natta ML Hallinan E Kowdley KV Abdelmalek M. Vibration-controlled transient Elastography to assess fibrosis and steatosis in patients with nonalcoholic fatty liver disease. Clin Gastroenterol Hepatol. (2019) 17:156–63.e2. doi: 10.1016/j.cgh.2018.04.043, PMID: 29705261 PMC6203668

[13] Kim D Cholankeril G Loomba R Ahmed A. Prevalence of fatty liver disease and fibrosis detected by transient Elastography in adults in the United States, 2017-2018. Clin Gastroenterol Hepatol. (2021) 19:1499–501.e2. doi: 10.1016/j.cgh.2020.08.017, PMID: 32801011

[14] Singh S Allen AM Wang Z Prokop LJ Murad MH Loomba R. Fibrosis progression in nonalcoholic fatty liver vs nonalcoholic Steatohepatitis: a systematic review and Meta-analysis of paired-biopsy studies. Clin Gastroenterol Hepatol. (2015) 13:643–654.e9. doi: 10.1016/j.cgh.2014.04.014, PMID: 24768810 PMC4208976

[15] Lorbeer R Bayerl C Auweter S Rospleszcz S Lieb W Meisinger C. Association between Mri-derived hepatic fat fraction and blood pressure in participants without history of cardiovascular disease. J Hypertens. (2017) 35:737–44. doi: 10.1097/hjh.0000000000001245, PMID: 28253218

[16] VanWagner LB Wilcox JE Colangelo LA Lloyd-Jones DM Carr JJ Lima JA. Association of Nonalcoholic Fatty Liver Disease with subclinical myocardial remodeling and dysfunction: a population-based study. Hepatology. (2015) 62:773–83. doi: 10.1002/hep.27869, PMID: 25914296 PMC4549239

[17] Musso G Gambino R Tabibian JH Ekstedt M Kechagias S Hamaguchi M. Association of non-Alcoholic Fatty Liver Disease with chronic kidney disease: a systematic review and Meta-analysis. PLoS Med. (2014) 11:e1001680. doi: 10.1371/journal.pmed.1001680, PMID: 25050550 PMC4106719

[18] Valbusa F Bonapace S Agnoletti D Scala L Grillo C Arduini P. Nonalcoholic fatty liver disease and increased risk of 1-year all-cause and cardiac hospital readmissions in elderly patients admitted for acute heart failure. PLoS One. (2017) 12:e0173398. doi: 10.1371/journal.pone.0173398, PMID: 28288193 PMC5348001

[19] Sorrentino P Terracciano L D'Angelo S Ferbo U Bracigliano A Vecchione R. Predicting fibrosis worsening in obese patients with Nash through parenchymal fibronectin, Homa-Ir, and hypertension. Am J Gastroenterol. (2010) 105:336–44. doi: 10.1038/ajg.2009.587, PMID: 19861959

[20] Aneni EC Oni ET Martin SS Blaha MJ Agatston AS Feldman T. Blood pressure is associated with the presence and severity of nonalcoholic fatty liver disease across the Spectrum of Cardiometabolic risk. J Hypertens. (2015) 33:1207–14. doi: 10.1097/hjh.0000000000000532, PMID: 25693058

[21] Ekstedt M Franzén LE Mathiesen UL Thorelius L Holmqvist M Bodemar G. Long-term follow-up of patients with Nafld and elevated liver enzymes. Hepatology. (2006) 44:865–73. doi: 10.1002/hep.21327, PMID: 17006923

[22] Horie Y Suzuki A Kataoka E Sasaki T Hamada K Sasaki J. Hepatocyte-specific Pten deficiency results in Steatohepatitis and hepatocellular carcinomas. J Clin Invest. (2004) 113:1774–83. doi: 10.1172/jci20513, PMID: 15199412 PMC420505

[23] Allen AM Therneau TM Larson JJ Coward A Somers VK Kamath PS. Nonalcoholic fatty liver disease incidence and impact on metabolic burden and death: a 20 year-community study. Hepatology. (2018) 67:1726–36. doi: 10.1002/hep.29546, PMID: 28941364 PMC5866219

[24] Käräjämäki AJ Bloigu R Kauma H Kesäniemi YA Koivurova OP Perkiömäki J. Non-alcoholic fatty liver disease with and without metabolic syndrome: different long-term outcomes. Metabolism. (2017) 66:55–63. doi: 10.1016/j.metabol.2016.06.009, PMID: 27423871

