# Supplementary material for: Impact of hypertension on liver fibrosis in patients with metabolic dysfunction-associated fatty liver disease
Source: Front Med (Lausanne). 2025 Jan 22;12:1539283. doi: 10.3389/fmed.2025.1539283 (PMC11794791; doi:10.3389/fmed.2025.1539283)
Supplement: Supplementary file 1 [file Data_Sheet_1.docx]

**Impact of Hypertension on Liver Fibrosis in Patients with Metabolic Dysfunction-Associated Fatty Liver Disease**

**Zhifeng Gao^1^, Huan Deng^2^, Bowen Qin^2^, Liang Bai^1^, Jiangwei Li^1^, Jian Zhang^1,*^**

**Table of Contents**

**Supplementary Tables**

Supplementary Table1. Demographic and clinical characteristics of the participants according to the blood pressure categories.

Supplementary Table2. Demographic and clinical characteristics of the participants according to the Presence of NAFLD and Hypertension.

Supplementary Table1. Demographic and clinical characteristics of the participants according to the blood pressure categories.

| Characteristics | Non-hypertension (n=2511) | Elevated (n=861) | Stage 1 (n=1269) | Stage 2 (n=1326) | *P* |
| --- | --- | --- | --- | --- | --- |
| Age (years) | 42.17(40.83-43.5) | 51.69(49.25-54.12) | 48.66(46.85-50.47) | 57.94(56.15-59.73) | 0.00 |
| Sex, n (%) | 42.08% | 14.43% | 21.27% | 22.22% | 0.00 |
| Male | 41.21% | 68.69% | 54.62% | 52.71% |  |
| Female | 58.79% | 31.31% | 45.38% | 47.29% |  |
| Race/ethnicity, n (%) |  |  |  |  | 0.00 |
| Mexican American | 9.07% | 7.77% | 7.33% | 6.77% |  |
| Other Hispanic | 7.63% | 8.22% | 6.03% | 7.14% |  |
| Non-Hispanic White | 66.18% | 67.41% | 64.87% | 61.66% |  |
| Non-Hispanic Black | 9.08% | 8.19% | 10.98% | 17.55% |  |
| Other Race - Including Multi-Racial | 8.03% | 8.40% | 10.79% | 6.89% |  |
| Education level, n (%) |  |  |  |  | 0.00 |
| Less than high school | 7.48% | 9.49% | 9.29% | 13.00% |  |
| High school or equivalent | 24.05% | 26.92% | 29.65% | 30.39% |  |
| College or above | 68.46% | 63.59% | 61.05% | 56.61% |  |
| Family poverty income ratio | 2.58(2.55-2.62) | 2.65(2.59-2.7) | 2.63(2.58-2.68) | 2.53(2.49-2.57) | 0.02 |
| Married, % | 59.44% | 63.65% | 67.36% | 62.15% | 0.00 |
| Diabetes, n (%) |  |  |  |  | 0.00 |
| Yes | 9.77% | 13.99% | 15.00% | 25.29% |  |
| No | 90.23% | 86.01% | 85.00% | 74.71% |  |
| Smoking status, n (%) |  |  |  |  | 0.00 |
| Current | 17.02% | 13.61% | 17.84% | 17.67% |  |
| Never | 60.80% | 54.04% | 52.37% | 49.80% |  |
| Former | 22.18% | 32.35% | 29.79% | 32.52% |  |
| Weekly minutes | 1389.47(1271.49-1507.46) | 1434.01(1230.79-1637.24) | 1268.21(1148.91-1387.51) | 1040.45(939.34-1141.56) | 0.02 |
| BMI | 28.1(27.63-28.57) | 29.02(28.37-29.67) | 30.82(30.26-31.37) | 30.05(29.49-30.62) | 0.00 |
| Median stiffness (kPa) | 5.46(5.22-5.71) | 6.04(5.48-6.6) | 6.48(6.04-6.91) | 6.22(5.77-6.66) | 0.00 |
| Significant Fibrosis (%) | 4.32% | 6.42% | 9.33% | 10.70% | 0.00 |
| Advanced Fibrosis (%) | 2.38% | 3.26% | 5.14% | 3.65% | 0.00 |
| Median CAP (dB/m) | 249.29(245.02-253.55) | 271.08(265.53-276.62) | 282.45(277.53-287.37) | 284.13(279.89-288.36) | 0.00 |
| <285 dB/m (%) | 72.14% | 61.72% | 50.25% | 51.53% | 0.00 |
| ≥285 dB/m (%) | 27.86% | 38.28% | 49.75% | 48.47% | 0.00 |
| Fasting glucose (mg/dL) | 5.77(5.7-5.84) | 6.17(5.97-6.37) | 6.18(6.08-6.27) | 6.54(6.38-6.7) | 0.00 |
| HbA1c (%) | 5.5(5.47-5.53) | 5.71(5.61-5.81) | 5.75(5.71-5.79) | 5.99(5.88-6.09) | 0.00 |
| HDL-cholesterol (mg/dL) | 54.27(53.25-55.28) | 52.62(50.99-54.24) | 51.8(50.27-53.34) | 53.3(51.95-54.66) | 0.02 |
| LDL-cholesterol (mg/dL) | 106.91(104.6-109.22) | 106.09(102.02-110.16) | 115.76(112.5-119.02) | 112.37(108.64-116.1) | 0.00 |
| hs-CRP (mg/L) | 3.74(3.37-4.11) | 3.02(2.63-3.4) | 3.66(3.25-4.06) | 4.4(3.54-5.27) | 0.00 |
| ALT (U/L) | 20.07(19.36-20.78) | 23.12(22.06-24.17) | 26.32(24.84-27.81) | 24.02(22.62-25.41) | 0.00 |
| ALB (g/dL) | 4.13(4.1-4.15) | 4.16(4.13-4.19) | 4.12(4.09-4.14) | 4.07(4.04-4.1) | 0.00 |
| AST (U/L) | 20.26(19.69-20.82) | 21.66(21.01-22.32) | 22.68(21.91-23.45) | 22.31(21.55-23.07) | 0.00 |
| GGT (IU/L) | 23.3(21.95-24.64) | 30.36(26.33-34.39) | 34.27(32.08-36.45) | 33.93(31.32-36.53) | 0.00 |
| Total bilirubin (mg/dL) | 0.46(0.44-0.48) | 0.52(0.49-0.56) | 0.44(0.42-0.46) | 0.47(0.45-0.5) | 0.00 |
| Total cholesterol (mg/dL) | 183.49(180.32-186.65) | 184.54(179.43-189.64) | 196.48(192.77-200.19) | 193.11(189.1-197.12) | 0.00 |
| Triglycerides (mg/dL) | 127.09(119.6-134.58) | 143.67(135.07-152.27) | 158.57(148.92-168.22) | 152.47(145.9-159.04) | 0.00 |
| Uric acid (mg/dL) | 5.12(5.03-5.2) | 5.66(5.54-5.78) | 5.55(5.44-5.66) | 5.57(5.43-5.71) | 0.00 |

Supplementary Table2. Demographic and clinical characteristics of the participants according to the Presence of NAFLD and Hypertension

| Characteristics | Control  (n=1740) | Hypertension only (n=1912) | NAFLD only  (n=771) | NAFLD and Hypertension  (n=1544) | P |
| --- | --- | --- | --- | --- | --- |
| Age (years) | 40.41(38.83-41.99) | 52.48(50.73-54.22) | 46.71(44.93-48.49) | 52.58(50.91-54.26) | 0.00 |
| Sex, n (%) | 29.16% | 32.04% | 12.92% | 25.88% | 0.00 |
| Male | 38.69% | 52.88% | 47.74% | 63.99% |  |
| Female | 61.31% | 47.12% | 52.26% | 36.01% |  |
| Race/ethnicity, n (%) |  |  |  |  | 0.00 |
| Mexican American | 6.67% | 5.28% | 15.30% | 9.61% |  |
| Other Hispanic | 7.88% | 7.49% | 6.99% | 6.45% |  |
| Non-Hispanic White | 67.86% | 63.19% | 61.84% | 66.14% |  |
| Non-Hispanic Black | 9.55% | 15.34% | 7.87% | 8.78% |  |
| Other Race - Including Multi-Racial | 8.04% | 8.70% | 8.00% | 9.02% |  |
| Education level, n (%) |  |  |  |  | 0.00 |
| Less than high school | 7.03% | 10.34% | 8.66% | 10.79% |  |
| High school or equivalent | 22.61% | 27.67% | 27.78% | 30.81% |  |
| College or above | 70.36% | 61.99% | 63.56% | 58.40% |  |
| Family poverty income ratio | 2.59(2.55-2.63) | 2.59(2.55-2.63) | 2.57(2.51-2.62) | 2.61(2.57-2.66) | 0.29 |
| Married, % | 57.17% | 60.22% | 65.32% | 69.77% |  |
| Diabetes, n (%) |  |  |  |  | 0.00 |
| Yes | 3.68% | 11.32% | 25.55% | 25.92% |  |
| No | 96.32% | 88.68% | 74.45% | 74.08% |  |
| Smoking status, n (%) |  |  |  |  | 0.00 |
| Current | 17.16% | 17.56% | 16.65% | 15.45% |  |
| Never | 62.42% | 53.24% | 56.61% | 50.58% |  |
| Former | 20.42% | 29.21% | 26.74% | 33.97% |  |
| Weekly minutes | 1458.01(1312.82-1603.2) | 1266.77(1175.11-1358.44) | 1212(1061.01-1363) | 1211.82(1084.74-1338.91) | 0.00 |
| BMI | 25.9(25.52-26.28) | 27.4(27.06-27.75) | 33.8(33.09-34.5) | 33.16(32.59-33.74) | 0.00 |
| Median stiffness (kPa) | 4.8(4.67-4.92) | 5.22(5.05-5.4) | 7.18(6.42-7.94) | 7.49(7.09-7.9) | 0.00 |
| Significant Fibrosis (%) | 2.04% | 3.92% | 10.23% | 14.83% |  |
| Advanced Fibrosis (%) | 0.52% | 1.27% | 7.22% | 7.47% |  |
| Median CAP (dB/m) | 219.25(216.62-221.88) | 233.76(231.52-236) | 327.07(323.87-330.26) | 333.6(330.32-336.88) | 0.00 |
| Fasting glucose (mg/dL) | 5.49(5.42-5.56) | 5.88(5.81-5.94) | 6.52(6.38-6.66) | 6.78(6.58-6.98) | 0.00 |
| HbA1c (%) | 5.34(5.31-5.38) | 5.61(5.56-5.65) | 5.9(5.82-5.98) | 6.06(5.97-6.15) | 0.00 |
| HDL-cholesterol (mg/dL) | 57.16(55.83-58.49) | 57.12(55.91-58.32) | 46.78(46.04-47.52) | 47.14(46.11-48.17) | 0.00 |
| LDL-cholesterol (mg/dL) | 106.65(104.17-109.12) | 111.68(108.77-114.58) | 107.58(103.63-111.54) | 112.2(108.22-116.17) | 0.02 |
| hs-CRP (mg/L) | 3.01(2.57-3.44) | 3(2.48-3.51) | 5.65(4.82-6.48) | 4.56(4.21-4.91) | 0.00 |
| ALT (U/L) | 17.96(17.3-18.62) | 20.44(19.64-21.25) | 25.56(23.91-27.2) | 29.61(28.13-31.08) | 0.00 |
| ALB (g/dL) | 4.16(4.13-4.19) | 4.13(4.1-4.16) | 4.04(4-4.08) | 4.09(4.06-4.12) | 0.00 |
| AST (U/L) | 19.77(19.14-20.41) | 20.85(20.53-21.18) | 21.5(20.62-22.39) | 23.93(23.27-24.6) | 0.00 |
| GGT (IU/L) | 20.36(18.97-21.75) | 27.5(25.37-29.64) | 30.92(28.13-33.71) | 39.54(37.44-41.63) | 0.00 |
| Total bilirubin (mg/dL) | 0.48(0.46-0.5) | 0.48(0.45-0.5) | 0.43(0.4-0.46) | 0.47(0.44-0.5) | 0.05 |
| Total cholesterol (mg/dL) | 182.53(179.62-185.43) | 191.03(187.69-194.38) | 185.97(180.67-191.28) | 193.13(188.77-197.5) | 0.00 |
| Triglycerides (mg/dL) | 108.31(102.34-114.28) | 124.34(119.95-128.73) | 175.71(162.15-189.28) | 185.15(178.29-192.01) | 0.00 |
| Uric acid (mg/dL) | 4.87(4.79-4.95) | 5.3(5.21-5.38) | 5.75(5.57-5.93) | 5.92(5.81-6.04) | 0.00 |
